# Real-world use of ranibizumab for neovascular age-related macular degeneration in Taiwan

**DOI:** 10.1038/s41598-018-25864-0

**Published:** 2018-05-10

**Authors:** Yi-Sheng Chang, Wan-Ju Lee, Chen-Chee Lim, Shih-Hao Wang, Sheng-Min Hsu, Yi-Chian Chen, Chia-Yi Cheng, Yu-Ti Teng, Yi-Hsun Huang, Chun-Chieh Lai, Sung-Huei Tseng

**Affiliations:** 1 Department of Ophthalmology, College of Medicine, National Cheng Kung University, Tainan, Taiwan; 2 Department of Ophthalmology, National Cheng Kung University Hospital, College of Medicine, National Cheng Kung University, Tainan, Taiwan; 3 Institute of Clinical Pharmacy and Pharmaceutical Sciences, College of Medicine, National Cheng Kung University, Tainan, Taiwan; 4 Department of Ophthalmology, National Taiwan University Hospital, College of Medicine, National Taiwan University, Taipei, Taiwan; 5 Institute of Clinical Medicine, College of Medicine, National Cheng Kung University, Tainan, Taiwan

## Abstract

This study investigated the “real-world” use of ranibizumab for neovascular age-related macular degeneration (nAMD) in Taiwan and assessed the visual outcome. We reviewed the medical records at National Cheng Kung University Hospital, Taiwan, during 2012–2014 for 264 consecutive eyes of 229 patients with nAMD, who applied for ranibizumab covered by national health insurance. A total of 194 eyes (73.5%) in 179 patients (65.5% men; mean ± standard deviation age 69.4 ± 10.7 years) were pre-approved for treatment. Applications for treatment increased year by year, but approval rates decreased during this time. The major causes of rejection for funding were diseases mimicking nAMD, including macular pucker/epiretinal membrane, macular scarring, dry-type AMD, and possible polypoidal choroidal vasculopathy. After completion of three injections in 147 eyes, visual acuity significantly improved, gaining ≥1 line in 51.8% of eyes and stabilising in 38.3% of 141 eyes in which visual acuity was measured. The 114 eyes approved with only one application had a better visual outcome than the 27 eyes approved after the second or third applications. In conclusion, ranibizumab is effective for nAMD; however, approval after the second or third application for national health insurance cover is a less favourable predictor of visual outcome.

## Introduction

Age-related macular degeneration (AMD) is the leading cause of blindness in developed countries, and its risk factors include old age, white race, genetic susceptibility, and smoking^1,2^. In Taiwan, the prevalence of early AMD (drusen or retinal pigment epithelial abnormalities) is 15.0% in individuals aged ≥65 years and 16.9% in those aged ≥80 years, whereas the prevalence of late AMD (nAMD or geographic atrophy) is 7.3% in people aged ≥65 years and 11.4% in those aged ≥80 years^3^. Taiwan has one of the most rapidly aging populations in the world, with 12.5% of people aged ≥65 years in 2015. Further, it is estimated that 36.7% of the Taiwanese population will be aged ≥65 years by 2050, far exceeding the projected global figure of 12% by that time^4^. Clearly, it is crucial to facilitate health care for the elderly population with nAMD.

A major objective in the management of nAMD in the clinical setting is to halt or delay progression and, if possible, improve the status of the disease. Nowadays, the first-line treatment for nAMD is intravitreal injection of an anti-vascular endothelial growth factor (anti-VEGF) such as ranibizumab (Lucentis^®^; Genentech Inc., San Francisco, CA, USA; Novartis Pharma AG, Basel, Switzerland), which was approved by the US Food and Drug Administration in 2006, by the European Medicines Agency in 2007, and by the Taiwan Food and Drug Administration in 2009. The national health insurance program implemented by the Taiwanese government in 1995 and now covering 99% of the 23.5 million people residing in Taiwan, approved the conditional reimbursement of ranibizumab when used for nAMD since January 2011, provided that the application was approved in advance.

Although clinical trials are the gold standard for approval of a new medicine, use of ranibizumab to treat nAMD in everyday clinical practice^5–7^ is different from its use in clinical trials. Real-world data from Asia are scarce^8,9^. To fill this knowledge gap, we investigated the everyday use of ranibizumab in patients with nAMD, analysed the patterns of approval and rejection of applications to use the drug under the national health insurance coverage in Taiwan, and assessed the visual outcome.

## Patients and Methods

### Pre-approval of ranibizumab for use in nAMD by the national health insurance scheme in Taiwan

Patients in Taiwan with a diagnosis of nAMD are eligible for reimbursement of ranibizumab when the following requirements are met and the application is approved in advance of treatment: age 50 years or older; fluorescein angiography (FA) and optical coherence tomography (OCT) performed within the past month compatible with a diagnosis of nAMD; and best-corrected visual acuity (BCVA) within 0.05–0.5 (20/400-20/40)^10^.

Grounds for declining approval to use ranibizumab are as follows: macular scarring; subretinal fibrosis or geographic atrophy; a possible diagnosis of polypoidal choroidal vasculopathy (PCV) needing confirmation by indocyanine green angiography; and choroidal neovascularisation caused by other conditions, such as high myopia or angioid streaks^10^.

An application form, along with the patient’s medical records, representative images, and the patient’s consent are submitted electronically to the national health insurance system. After approval, three doses of ranibizumab per eye would be reimbursed. For applications that are rejected for the first round of treatment, the second-time or third-time applications as justified by the clinician could be submitted as part of an appeal process.

### Enrolment of study subjects

This retrospective study was approved by the Institutional Review Board at National Cheng Kung University Hospital, Taiwan (approval code A-ER-104-322), and was performed in accordance with the Declaration of Helsinki and in accordance with the data protection rules in Taiwan. The need for informed consent was waived because patient anonymity was ensured by the data source.

We reviewed the medical records of all patients who attended National Cheng Kung University Hospital from January 1, 2012 to December 31, 2014 with a diagnosis of nAMD and for whom applications were made to the national health insurance system for approval to use ranibizumab. Patients for whom applications were successful received intravitreal injections of ranibizumab 0.5 mg at a maximum of three doses per eye, ideally at an interval of 1 month apart. We recorded and analysed the patient age and sex data, laterality of the eyes that underwent treatment, initial BCVA, FA, and OCT at application, days taken for approval or rejection, reasons for rejection, dates of injection, and final BCVA.

### Outcome measurements

The primary outcome was the rate of approval of ranibizumab (granted by the national health insurance system). We analysed the factors potentially influencing the likelihood of approval versus rejection, including the profiles of patients/eyes, year and order of application, and the years of experience of the attending clinicians who diagnosed and treated the patients.

The secondary outcome was the difference in BCVA between before and after treatment. BCVA was assessed using the Landolt C chart and converted to logMAR (logarithm of the minimum angle of resolution) units. For analysis of visual outcome, patients were included if they had post-treatment BCVA measurements available and if they had acceptable intervals between injections defined as follows: the second dose injected approximately 1–2 months after the first dose; the third dose injected approximately 1–3 months after the second dose; and post-treatment BCVA measured approximately 1–4 months after the third injection. Patients who missed or inappropriately deferred treatment sessions were excluded from the analysis of visual outcome. Two groups of eyes were compared for visual outcome: those approved for funding on only one application (group 1) and those approved after 2–3 applications (group 2).

### Statistical analysis

The study data were entered into the Excel computer software program (Office 2010, Microsoft Corp., Redmond, WA, USA). Categorical variables were presented as number and percentage values and were analysed using the chi-squared test. Continuous variables presented with the mean, standard deviation (SD) and range, were analysed using the Student’s *t* -test. A paired Student’s *t* -test was used when the measurements were on the same eye over time. A *P*-value < 0.05 was deemed to be statistically significant.

## Results

**Figure 1:**
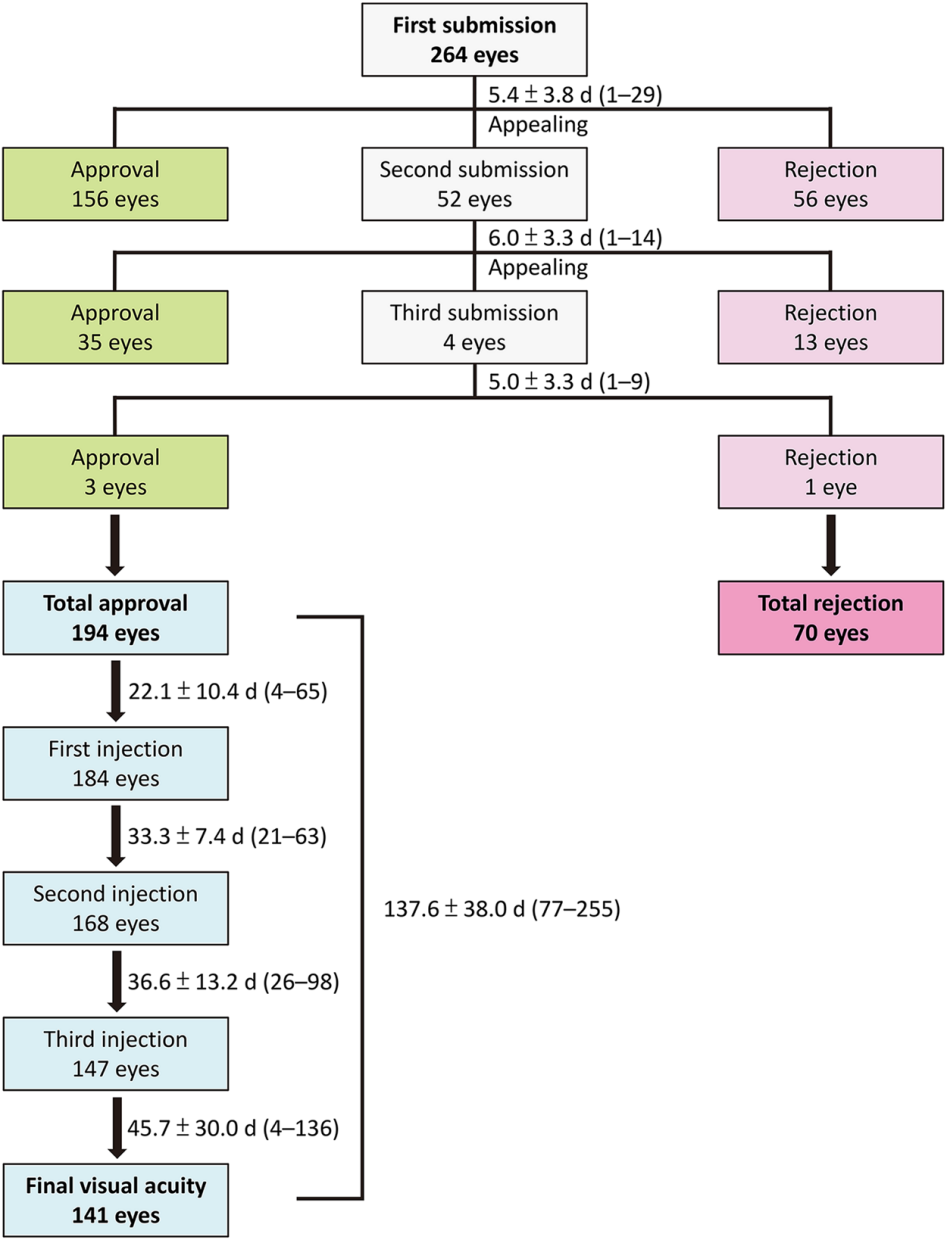
Flow chart of 264 eyes (229 patients) with neovascular age-related macular degeneration at a university medical centre in 2012–2014 for which an application was made to the national health insurance system in Taiwan for funding of treatment with ranibizumab.

In 2012–2014, applications to fund treatment using ranibizumab were made to the national health insurance system for 264 consecutive eyes of 229 patients, and approval was received for 194 eyes (73.5%) of 179 patients after 1–3 applications (see Fig. 1).

As shown in Table 1, 127 (65.5%) of the 194 eyes approved for treatment were in male patients. The mean ± SD patient age was 69.4 ± 10.7 (range 50–94) years (69.7 ± 10.5 [range 50–94] years for men and 68.8 ± 11.0 [range 51–89] years for women; *P* = 0.599). Simultaneous applications were made for bilateral treatment in 10 of 15 patients with both eyes approved, and applications were made on separate occasions for treatment of both eyes in the remaining 5 patients.

**Table 1:** Demographics and characteristics of 264 eyes (229 patients) with neovascular age-related macular degeneration in a university medical centre in 2012–2014 for which an application was made to the national health insurance system in Taiwan for funding of treatment with ranibizumab.

Characteristics	Eyes, n (%)		*P*-value
	Approval	Rejection	
	n = 194 (73.5%)	n = 70 (26.5%)	
Sex			0.637
Male	127 (72.6)	48 (27.4)	
Female	67 (75.3)	22 (24.7)	
Age, years			0.226
50–59	36 (85.7)	6 (14.3)	
60–69	61 (71.8)	24 (28.2)	
70–79	62 (68.9)	28 (31.1)	
80+	35 (74.5)	12 (25.5)	
Laterality			0.968
Right eye	102 (73.4)	37 (26.6)	
Left eye	92 (73.6)	33 (26.4)	
Year			0.003
2012	37 (92.5)	3 (7.5)	
2013	69 (75.8)	22 (24.2)	
2014	88 (66.2)	45 (33.8)	
Application order			
First-time	156 (59.1)	108 (40.9)	
Second-time (appealing)	35 (74.5)	12 (25.5)	
Third-time (appealing)	3 (75.0)	1 (25.0)	

**Figure 2:**
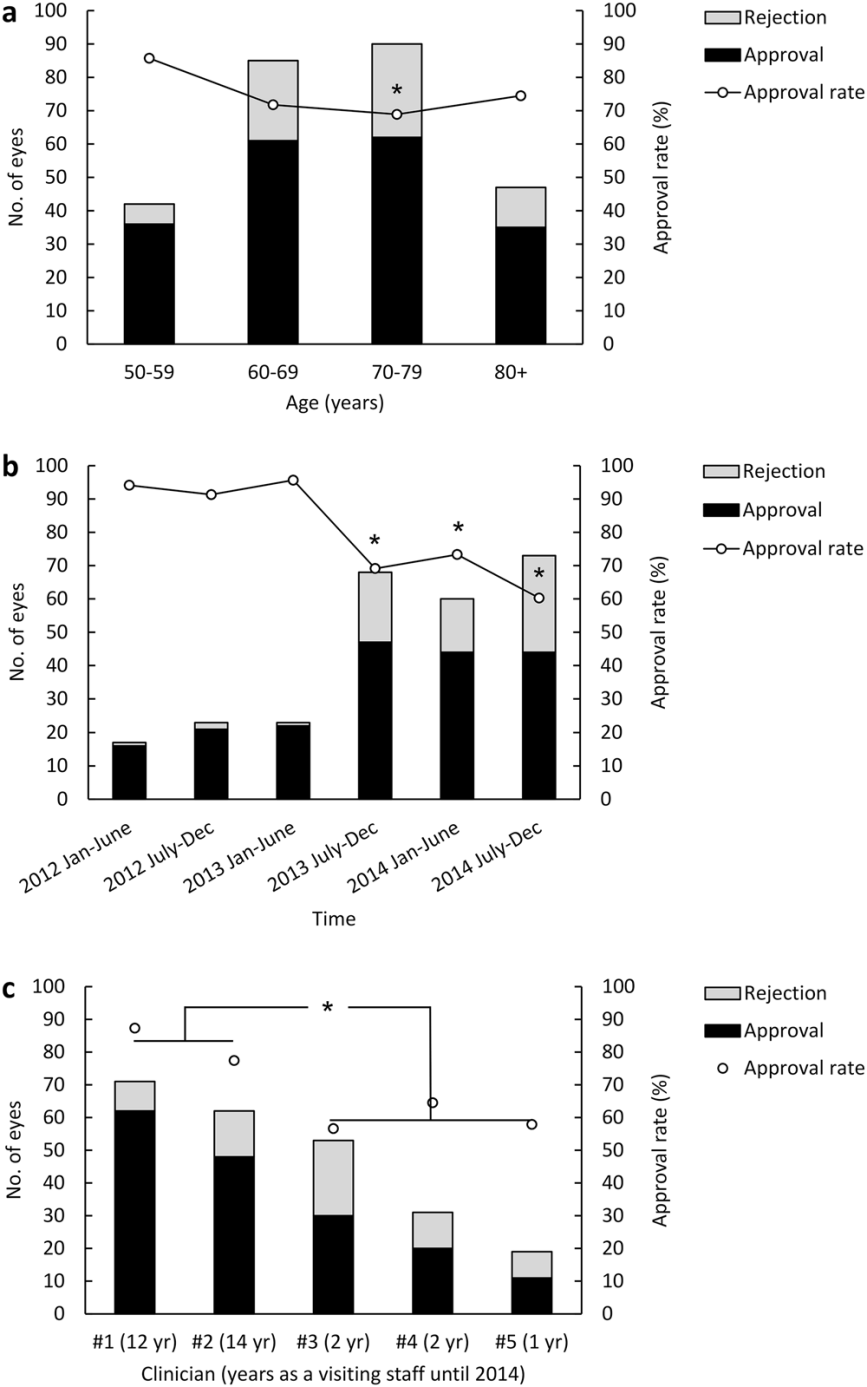
Distribution of 264 eyes (229 patients) with neovascular age-related macular degeneration treated at a university medical centre in 2012–2014 for which an application was made to the national health insurance system for funding of treatment with ranibizumab. (**a**) Distribution by age. *P < 0.05 versus 50–59 years. (**b**) Distribution by year and month at application. *P < 0.05 versus January to June 2012. (**c**) Distribution by clinician according to years of experience in ophthalmological practice. *P < 0.05; senior clinicians #1 and #2 versus junior clinicians #3, #4, and #5.

The lowest approval rate (68.9%) was for eyes in patients aged 70–79 years and the highest approval rate was in those aged 50–59 years (85.7%; *P* = 0.039; Table 1 and Fig. 2a). The number of eyes for which applications were made increased markedly after July 2013, but the approval rate has decreased since then (*P* = 0.003), as shown in Table 1 and Fig. 2b.

First-time applications were approved at a rate of 59.1%, which was substantially lower than the approval rates of second-time and third-time applications (74.5% and 75.0%, respectively, for eyes that were subject to an appeal by clinicians; Table 1).

The approval rates for applications made by the five clinicians treating the greatest numbers of patients with nAMD at our institution ranged from 56.6% to 86.1%; the approval rate varied significantly (*P* < 0.001) according to years of clinical experience (Fig. 2c).

The mean ± SD times taken to process first-time, second-time, and third-time applications were 5.4 ± 3.8 (range 1–29) days, 6.0 ± 3.3 (range 1–14) days, and 5.0 ± 3.3 (range 1–9) days, respectively; the differences were not statistically significant (Fig. 1). Further, there was no statistically significant difference in the time interval between approval and rejection of each application.

**Figure 3:**
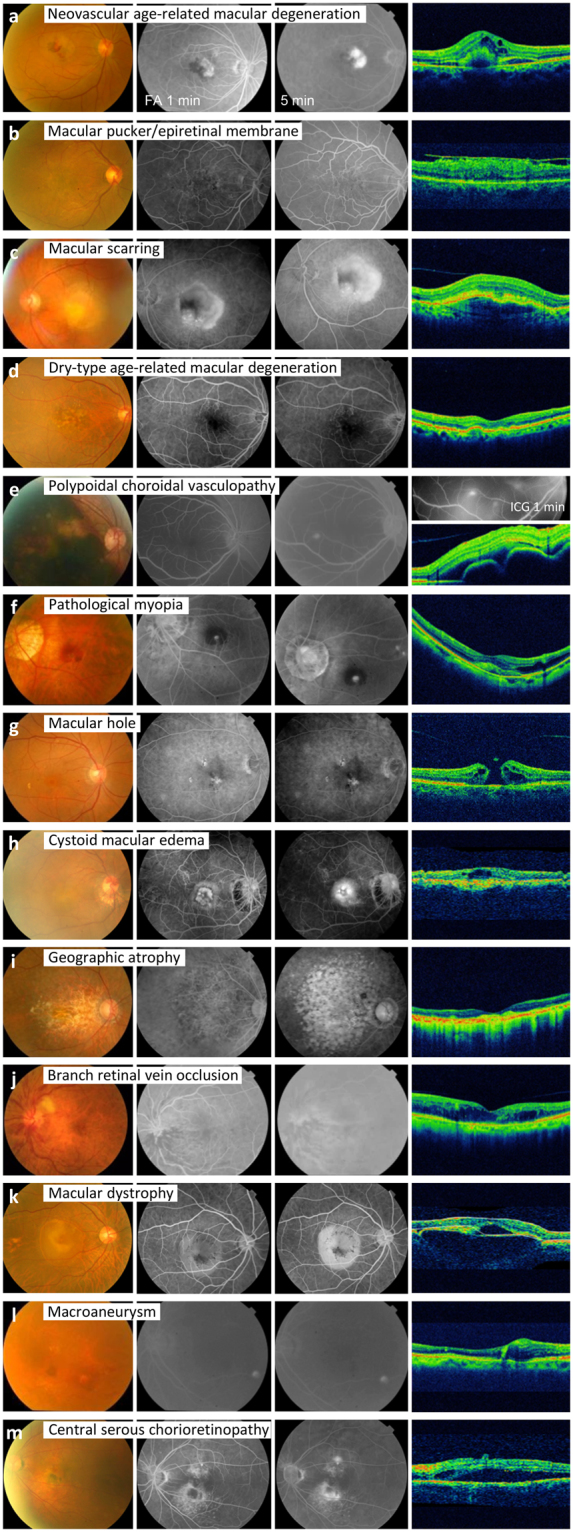
Representative fundus images for typical neovascular age-related macular degeneration (**a**) and other mimicking diseases that were rejected by the national health insurance system (**b–m**). FA, fluorescein angiography; ICG, indocyanine green.

Table 2 shows the reasons for rejection for 108 eyes of 95 patients at the first-time application for use of ranibizumab. The main reasons were presence of disease mimicking nAMD (88 eyes, comprising 81.5% of 108 rejected eyes), including macular pucker/epiretinal membrane (ERM; 23 eyes, comprising 21.3% of 108 rejected eyes), macular scarring (17 eyes, 15.7%), dry-type AMD (17 eyes, 15.7%), and possible PCV (14 eyes, 13.0%). Figure 3 shows images of eyes representative of the various disease mimics. Twenty (18.5%) of the eyes for which funding was declined were found to have technical issues associated with the application, mostly concerning accompanying images of poor quality and low resolution or absence of FA or OCT images, as was the case in 12 (11.1%) eyes. When the problem of the accompanying images was resolved, 9 eyes were approved on the second application and 1 eye was approved on the third application.

**Table 2:** Reasons for rejection in 108 eyes (95 patients) on first-time application for funding of ranibizumab by the national health insurance system.

Reasons for rejection	Eyes, n (%)
Diseases mimicking nAMD	88 (81.5)
Macular pucker/epiretinal membrane	23 (21.3)
Macular scarring	17 (15.7)
Dry-type AMD	17 (15.7)
Polypoidal choroidal vasculopathy	14 (13.0)
Pathological myopia	4 (3.7)
Macular hole	3 (2.8)
Cystoid macular oedema	3 (2.8)
Geographic atrophy	1 (0.9)
Central/branch retinal vein occlusion	1 (0.9)
Macular dystrophy	1 (0.9)
Macroaneurysm	1 (0.9)
Central serous chorioretinopathy	1 (0.9)
Administrative issues	20 (18.5%)
Poor quality, low resolution, or absence of FA/OCT	12 (11.1)
Corrected visual acuity <0.05	5 (4.6)
Corrected visual acuity >0.5	1 (0.9)
Inaccurate diagnosis in the operation consent	1 (0.9)
Misfiling the fellow normal eye	1 (0.9)

AMD, age-related macular degeneration; FA, fluorescein angiography: nAMD, neovascular age-related macular degeneration; OCT, optical coherence tomography.

Figure 1 shows the “real-world” timeline of ranibizumab injections in the 194 eyes in 179 patients approved for treatment in this study. Three injections were administered to 147 (75.8%) eyes within the designated appropriate intervals, two were administered to 21 (10.8%) eyes, one to 16 (8.2%) eyes, and none to 10 (5.2%) eyes.

Table 3 showed the patient demographics and characteristics for 141 eyes analysed for visual outcome versus the remaining 53 eyes that were excluded because of missed or inappropriately deferred treatment sessions or non-recorded final visual acuity.

**Table 3:** Demographics and characteristics of 194 eyes (179 patients) with neovascular age-related macular degeneration treated at a university medical centre in 2012–2014 for which ranibizumab was approved before treatment by the national health insurance system in Taiwan.

Characteristics	Eyes, n (%)		*P*-value
	Final visual acuity analysed	Not analysed^a^	
	n = 141 (72.7%)	n = 53 (27.3%)	
Sex			0.023
Male	99 (78.0)	28 (22.0)	
Female	42 (62.7)	25 (37.3)	
Age, years			0.666
50–59	29 (80.6)	7 (19.4)	
60–69	43 (70.5)	18 (29.5)	
70–79	45 (72.6)	17 (27.4)	
80+	24 (68.6)	11 (31.4)	
Laterality			0.491
Right eye	72 (70.6)	30 (29.4)	
Left eye	69 (75.8)	23 (24.2)	
Year			0.524
2012	25 (67.6)	12 (32.4)	
2013	49 (71.0)	20 (29.0)	
2014	67 (76.1)	21 (23.9)	
Application order			0.802
First-time	114 (73.1)	42 (26.9)	
Second- or third-time	27 (71.1)	11 (28.9)	

^a^Due to missed or inappropriately deferred treatment sessions or to non-recorded final visual acuity.

**Figure 4:**
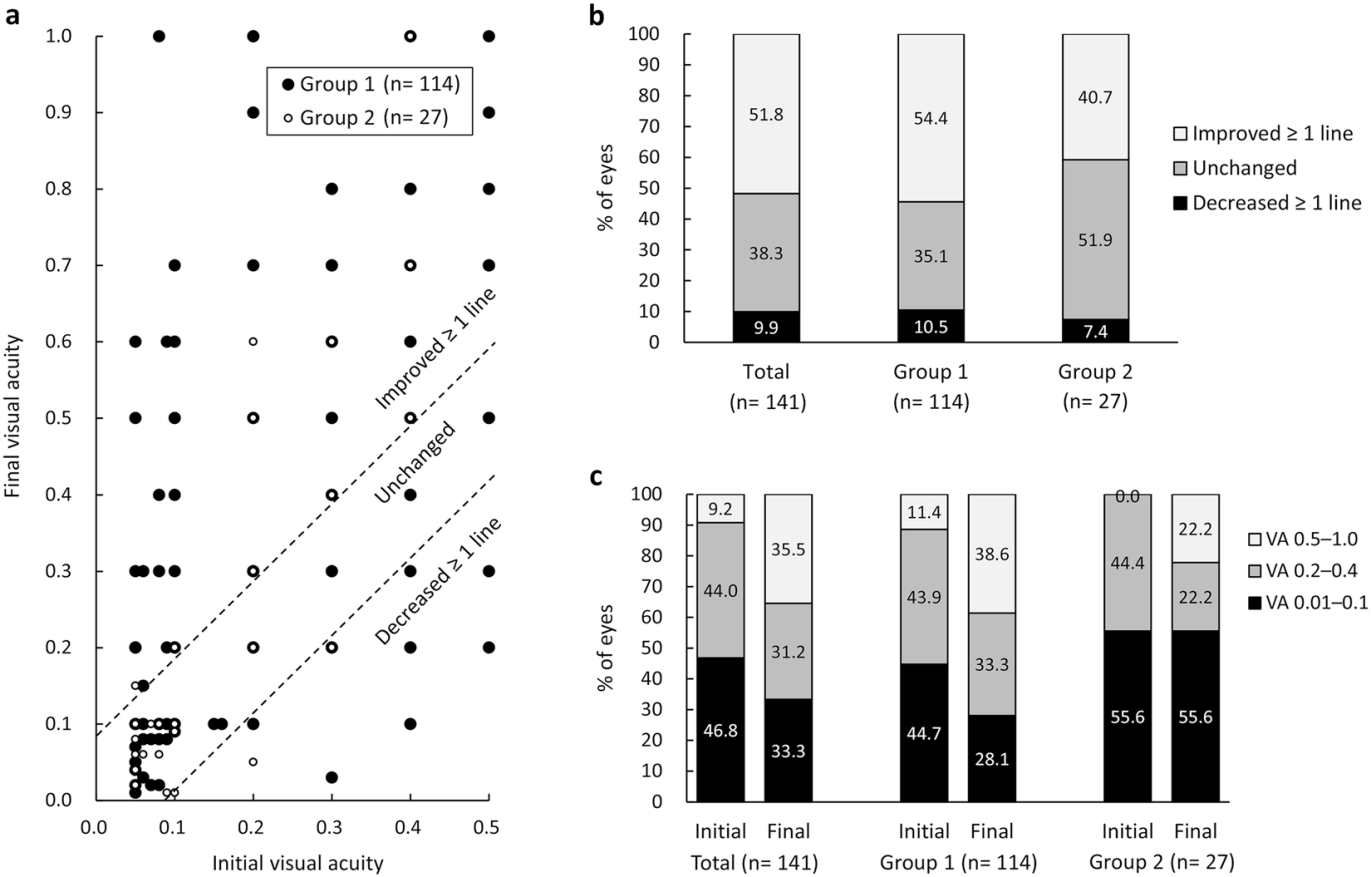
Initial versus final visual acuity in 141 eyes with documented visual acuity before and after treatment with ranibizumab. Group 1 comprises the eyes approved by the national health insurance system in Taiwan on only one application. Group 2 comprises the eyes approved on the second application (26 eyes) or third application (1 eye). (**a**) Scatter plot of initial versus final visual acuity. (**b**) Categorisation of visual improvement. (**c**) Categorisation of visual acuity as good (0.5–1.0), fair (0.2–0.4), and poor (0.01–0.1).

In the visual outcome analysis, the mean visual acuity (VA) in the 141 eyes that received three injections was 0.804 ± 0.349 logMAR (equivalent to 0.157 on the Landolt C chart) at baseline and 0.653 ± 0.486 (equivalent to 0.222 on the Landolt C chart) after the injections (*P* < 0.001; Table 4). The 114 eyes in group 1 (approved on only one application) had significant visual improvement after treatment (*P* < 0.001), but in group 2, the 26 eyes approved on the second application and the 1 eye approved on the third application did not (*P* = 0.780). There was no statistically significant difference in initial VA between the two groups (*P* = 0.116), but the final VA was significantly better in group 1 than in group 2 (*P* = 0.020). Figure 4a shows the distribution of initial versus final VA; visual outcome was improved in 73 (51.8%) of 141 eyes, was unchanged in 54 (38.3%) eyes, and was decreased in 14 (9.9%) eyes (Fig. 4b). Compared with group 1, group 2 tended to have a smaller proportion of eyes with visual improvement of ≥1 line but the difference was not statistically significant (Fig. 4b). Unlike group 1, group 2 had the same proportion of eyes (55.6%) with poor VA (0.01–0.1) after treatment (Fig. 4c).

**Table 4:** Initial versus final visual acuity in 141 eyes with documented visual acuity before and after treatment with ranibizumab. Group 1 includes the eyes approved by the national health insurance system on only one application. Group 2 includes the eyes approved on the second application (n = 26) or third application (n = 1).

Visual acuity	Total (n = 141)		Group 1 (n = 114)		Group 2 (n = 27)	
(logMAR)	Initial	Final	Initial	Final	Initial	Final
Mean	0.804	0.653	0.782	0.600	0.896	0.876
(Landolt C equivalent)	(0.157)	(0.222)	(0.165)	(0.251)	(0.127)	(0.133)
SD	0.349	0.486	0.352	0.459	0.328	0.542
*P*-value	<0.001		<0.001		0.780	

logMAR, logarithm of the minimum angle of resolution; SD, standard deviation.

Forty (31.3%) of the 128 eyes with an initial visual acuity <0.5 regained driving level vision (defined as visual acuity ≥0.5) after three injections. However, 2 (18.2%) of the 13 eyes with an initial visual acuity ≥0.5 lost their driving level vision after three injections (one decreased from 0.5 to 0.2 and the other from 0.5 to 0.3). None of the patients developed endophthalmitis or retinal detachment during the study period.

## Discussion

To our knowledge, this is the first report on use of ranibizumab for nAMD in Taiwan. We found that the final approval rate of ranibizumab applications was 73.5% (194/264) and that the decision made by the national health insurance system was influenced by patient age, year of application, order of application, and the seniority or years of clinical experience of the attending clinician. Of the 108 eyes (40.9%) for which the first application was rejected, 81.5% of cases were related to mimicking diseases, such as macular pucker/ERM, macular scarring, dry-type AMD, and possible PCV. Of note, 24.2% (47/194) of eyes approved for treatment with ranibizumab were undertreated because of missed or inappropriately delayed appointments. For the 75.8% of patients who received three injections at acceptable intervals, VA improved by ≥1 line in 51.8% and stabilised in 38.3%. The eyes approved with only one application had a better visual outcome than those approved on the second or third application.

Unlike the female predominance in most of the clinical trials, 65.5% of our patients with eyes approved for treatment with ranibizumab were men (compared with 50.1% in ANCHOR, 39.2% in CATT, 40.0% in IVAN, 35.2% in MARINA, and 40.2% in PIER)^11–15^. Male patients accounted for 36.7% (range 20.0–47.0%) in a meta-analysis of real-world studies collected from continental Europe, the UK, the USA, Canada, and Australia^16^. However, two epidemiological studies in Taiwan reported any stage of nAMD to be more prevalent in men (54.2% in the Puzih study and 64.1% in the Shihpai study)^3,17^. Our study population was drawn from Tainan, a subtropical region located at 22°59′ North, where older men have traditionally worked as farmers and been exposed to long hours of sunlight since their 20 s. These geographic and working characteristics would have contributed to the male predominance in the present study, and explain the younger mean age of our subjects (69.4 years versus 76.0–80.1 years in the major clinical trials^11–15^ and 72.7–81.7 years [overall mean 78.8 years] in representative real-world studies^16^).

In our study, the number of applications increased markedly after July 2013, which is attributed to increased public awareness of nAMD, more referrals from local medical practitioners, and the number of young retinal clinicians employed at our institution. However, the approval rate has decreased since July 2013, which is attributed to the increased numbers of patients with comorbid or confounding conditions and the relative inexperience of junior staff. When an application prior to treatment is set up under the health insurance system, diseases mimicking nAMD or at an advanced stage (such as macular scarring or geographic atrophy) would be excluded from treatment. Since the approval of a case indicates consensus between clinicians and reviewers, this mechanism has the advantages of facilitating therapeutic effectiveness and decreasing the number of unnecessary treatments and the burden of medical care. However, there are also the disadvantages of the application being time-consuming and the fact that the appeal process takes 1 week to 1 month to complete after the diagnosis. Such an approval needs to be obtained prior to the following ocular treatments covered by the national health insurance system in Taiwan: (1) ranibizumab or aflibercept (Eylea^®^, Bayer Pharma AG, Berlin, Germany) used for nAMD, macular oedema caused by diabetes mellitus or retinal vein occlusion, or choroidal neovascularisation caused by high myopia; (2) photodynamic therapy with verteporfin (Visudyne^®^, Novartis Pharma AG) used for PCV; (3) dexamethasone intravitreal implant (Ozurdex^®^, Allergan, Irvine, CA, USA) used for intractable non-infectious posterior uveitis or for macular oedema caused by central retinal vein occlusion; (4) cyclosporine ophthalmic emulsion (Restasis^®^, Allergan) used for dry eye-related keratopathy; and (5) cataract surgery for those aged <55 years. In this study, we only enrolled patients who received ranibizumab and not those who received aflibercept because aflibercept was not approved for reimbursement in the indication of nAMD by the national health insurance system in Taiwan until August 2014. By the end of 2014, very few patients at our institution had started treatment with aflibercept. Bevacizumab is still used off-label for nAMD in Taiwan so is not reimbursed by the national health insurance system and was not included either.

In this study, 108 (40.9%) of 264 eyes were rejected on their first application; of these, 81.5% involved up to 12 diseases mimicking nAMD. Not all of these differential disease patterns were typical; some were confounding or even mixed (e.g., nAMD with ERM), leading to diagnostic difficulty and rejection on the first application. Further, 12 (11.1%) of the rejected eyes had accompanying images of poor quality and low resolution or absence of FA or OCT images, which tended to be resolved (in 10 of 12 eyes, 83.3%) by providing higher resolution or additional images, achieving a 74.5–75.0% approval rate on the second or third application.

VA in patients with untreated nAMD has been reported to decline by 2–3 lines per year during the natural course of the disease^14,15,18^. This study showed that three injections achieved visual improvement of ≥1 line (equivalent to 5 EDTRS letters) in 51.8% of eyes and stabilisation within 1 line in 38.3% of eyes, which is comparable with the improvement of 3–10 EDTRS letters seen in the major clinical trials after the first three loading doses^11–15^. Our visual outcome is also comparable with that in a meta-analysis of real-world studies, in which the mean visual improvement at one year was 3.5 EDTRS letters after an average of 5.4 injections on a pure “*pro re nata*” (PRN; meaning as needed) regimen and 8.8 EDTRS letters after an average of 7.3 injections by a “treat-and-extend” regimen^16^. Although this study did not speculate on a long-term effect, a final VA of ≥0.5 was found in 35.5% of eyes at follow-up 1–4 months after the third injection in this study, which is slightly inferior to the 38.6% measured at one year after 12 monthly injections in the ANCHOR clinical trial^11^. However, a final VA ≤0.1 was observed in 33.3% of eyes, which is far more than the 16.4% in the ANCHOR clinical trial. This disparity is explained by the fact that the maximal effect occurred after 3–6 monthly doses in the clinical trials^11–15^ and that the three initial doses used in the current study still did not achieve a maximal effect. Another reason is the inclusion of more patients with diseases mimicking or confounding nAMD in group 2. Of note, a decline in vision of ≥1 line was seen in 9.9% of our subjects, who may need more injections or alternative or combination treatment^19^. Previous studies showed that the factors predicting less visual improvement include male sex, older age, better baseline VA, a larger neovascularisation area, a classic type of choroidal neovascularisation, geographic atrophy, a thicker central retina, detachment of retinal pigment epithelium, vitreomacular adhesion, and undertreatment^9,20–22^. The current study indicates that approval after the second or third application for insurance cover is a less favourable predictor of visual outcome.

The regimens used in the clinical registration trials for anti-VEGFs in nAMD are generally standardised, usually as either “monthly injections”, “treatment and PRN”, or “treatment and extended”^11–15^. However, in the “real-world” clinical setting, various regimens are used^7,23–25^. First, clinical trials usually enrol highly selected subjects with “pure” and naïve nAMD without comorbid or confounding diseases and without prior treatment, thereby ensuring comparability between treated and non-treated patients. In contrast, real-world settings involve a large and diverse group of patients with nAMD who may present additional manifestations of the disease at different stages (such as drusen, macular scarring, or geographic atrophy) and have confounding diseases or prior/concurrent/adjunctive treatment (such as other anti-VEGF agents, photocoagulation, or photodynamic therapy). These disease variations and individualised therapies may lead to less consistent and less satisfactory therapeutic effectiveness^26^.

Second, decision-making about treatment differs between the trial setting and actual clinical practice. In the clinical trials, treatment is started in eligible subjects after screening, and participants then receive sufficient treatment during the study, e.g., for 2 years^14,27–32^. However, treatment decisions in the real world are greatly affected by a host of factors in the health care system, including cost, geographic availability, ease of access, and particularly self-payment or reimbursement by third party payers^23^, usually leading to undertreatment and suboptimal therapeutic effectiveness^19,33–35^.

Third, participants in the trials are scheduled on an ideal and strict basis (i.e., monthly assessment and/or treatment), are reminded to attend for appointments by research assistants, and are covered for transport fees by sponsors. These factors would maximise patient compliance and minimise the possibility of undertreatment. However, patients in the real world, including 24.2% of our subjects, may delay or miss treatments or visits, often because of concomitant systemic disease, transport or ambulatory difficulties, unavailability of an accompanying family member, or financial factors^36,37^. This is supported by a study of US Medicare beneficiaries showing that 71.0% of patients discontinued treatment and 42.1% discontinued visits within 24 months of the initial treatment^38^.

Fourth, VA in the clinical trials is generally expressed as EDTRS letters, which increases accuracy and strengthens the statistical significance of the results of treatment. However, VA in the real-world setting is measured by the Landolt C or Snellen E chart for convenience, which may not be as sensitive for detecting differences in outcome. Recently, the International Consortium for Health Outcomes Measurement recommended a set of standardized patient-centred outcome measures for macular degeneration, including visual functioning and vision-related quality of life (distance VA, mobility and independence, emotional well-being, and reading and assessing information), disutility of care (number of treatments and complications of treatment), and disease control (presence of fluid, oedema, or haemorrhage)^39^. Such a comprehensive assessment should be very helpful to better evaluate patients’ outcomes.

To improve the therapeutic effectiveness of ranibizumab in patients with nAMD in real-world clinical practice, clinicians need to be well trained in diagnostic imaging, should provide health education and be able to provide therapeutic instructions to patients, and adhere to three monthly loading doses and subsequent maintenance doses. Patients are expected to be aware of their disease course and prognosis, use the Amsler chart for self-monitoring^40^, and comply with long-term treatment and follow-up appointments. Support, encouragement, and attendance of family members at appointments are also important for improving patients’ adherence with treatment. The government is expected to develop optimal public health policies and education in order to screen for or detect nAMD at its early stage. Early treatment affords the best chance of recovery and reduces unnecessary burden on the patient and society.

Given that a major part of decision-making about treatment in Taiwan relies on the reimbursement policies of the national health insurance scheme, this study raises two issues regarding eligibility for funded treatment with ranibizumab. First, VA is regulated in the range of 0.05–0.5. Eyes with a VA > 0.5, which may represent early disease and be curable, are excluded from reimbursement. Lee *et al*. reported the benefits of initiating ranibizumab therapy for nAMD in eyes with a baseline VA > 0.5 because these eyes maintained a better level of vision than those with a baseline VA < 0.5 at all time points for at least 2 years^41^. In contrast, a VA < 0.05 may be caused by comorbid ocular disease, such as amblyopia, corneal opacity, cataract, posterior capsular opacity following cataract surgery, vitreous opacity, advanced drusen, macular pucker/ERM, macular haemorrhage, or glaucoma. The nAMD in these eyes still needs treatment but is not eligible for reimbursement. Second, three ranibizumab injections per eye are funded and may be extended for a further four doses (i.e., seven lifetime doses per eye) if the initial three doses are shown to improve the patient’s VA and/or retinal morphology on OCT. Indeed, some of our patients later needed 4–6 loading doses to stabilise or improve their disease status, as in the clinical trials^11–15^. Although 38.3% of our patients showed no change in VA and 9.9% actually had a decreased VA after three doses, discontinuation of treatment may worsen vision more rapidly in these patients^14,15,42^. To provide better health care for our increasingly elderly population, more comprehensive reimbursement should be considered at the government level.

Our study has some limitations. First, we included only patients with nAMD fulfilling the reimbursement criteria set down by the national health insurance system. Exclusion of patients with less or more severe disease and those who self-funded their treatment led to underestimation of the population served by our hospital. Self-funded patients might be of a higher socioeconomic status, leading to selection bias. Second, our subjects were enrolled from only one medical centre, and there might be some differences in the real-world use of ranibizumab between different regions and between hospitals. Third, information obtained by retrospective review of medical records, as in our study, is likely to be incomplete. Fourth, the final visual acuity of our patients was not recorded at standard time intervals after the final injection. However, in spite of these shortcomings, we believe that our results provide important information about use of ranibizumab in the “real-world” in Taiwan. This study contributes to our knowledge regarding the patient characteristics, the application process, treatment scheduling, and therapeutic effectiveness of ranibizumab, and should help clinicians to evaluate and manage nAMD better. Finally, national strategies for use of ranibizumab and other anti-VEGF treatment can now be optimised.

## Conclusions

From our review of 264 eyes with nAMD for which applications were made to the national health insurance system in Taiwan to allow treatment with ranibizumab, the overall final approval rate was 73.5% in 2012–2014, but approvals have decreased since July 2013. In approved eyes, only 75.8% were treated with three injections of ranibizumab at acceptable intervals. VA was improved in 51.8% of patients and stabilised in 38.3%. Of note, the eyes approved on only one application had a better visual outcome than those approved on second or third applications. Hence, the number of applications for approval acts as a predictor of visual outcome. In order to facilitate the approval process and therapeutic effectiveness, we suggest accurate diagnostic imaging and document preparation, and more professional and administrative training. The long-term results of further injections warrant investigation in the future.
